# *Mycobacterium avium* subsp. *paratuberculosis* Infection in a Patient with HIV, Germany

**DOI:** 10.3201/eid0807.010388

**Published:** 2002-07

**Authors:** Elvira Richter, Johannes Wessling, Norbert Lügering, Wolfram Domschke, Sabine Rüsch-Gerdes

**Affiliations:** * National Reference Center for Mycobacteria, Borstel, Germany; † University of Münster, Münster, Germany

**Keywords:** *Mycobacterium avium* subsp. *paratuberculosis*, HIV, *Mycobacterium avium* complex, Johne disease

## Abstract

*Mycobacterium avium* subsp. *paratuberculosis* (MAP), the causative agent of Johne disease in ruminants, has been incriminated as the cause of Crohn disease in humans. We report the first case of human infection with MAP in a patient with HIV; infection was confirmed by obtaining isolates from several different specimen types.

Opportunistic infections caused by various *Mycobacterium* species are among the leading AIDS indicator diseases in HIV-positive patients (1). Infections with nontuberculous mycobacteria occur mainly in patients who have low CD4+ counts (<50 cells) or high virus counts (2); *Mycobacterium avium* complex is the most important mycobacterial species. *M. avium* complex includes the species *M. avium* and *M. intracellulare*, with *M. avium* consisting of *M. avium* subsp. *avium*, *M. avium* subsp. *sylvaticum,* and *M. avium* subsp. *paratuberculosis* (MAP). All these subspecies have identical 16S rRNA gene and 16S to 23S transcribed spacer sequences, as well as shared biochemical characteristics (3). However, MAP is dependent on mycobactin for its growth, whereas *M. avium* grows well on different solid media.

MAP is the causative agent of Johne disease, a chronic granulomatous ileitis occurring mainly in ruminants (4). MAP has been incriminated as the cause of Crohn disease in humans (5,6), although conflicting findings have been reported. However, culture-confirmed cases of MAP in human specimens remain rare (5,6).

## Case Report

A 36-year-old HIV-positive man, who had been treated at our hospital since 1995 for HIV, hepatitis C, and hemophilia, had profuse diarrhea (6–8 episodes/day), fever as high as 39.9°C, and 10 kg of body weight loss in 5 weeks. Laboratory findings included hemoglobin 9.6 g/dL, pseudocholinesterase 2,099 U/L, HIV-DNA virus count 500 copies/mL, CD4+ lymphocyte count 29 x 10^6^/mL, and C-reactive protein 76 mg/L. Stained colon tissue samples, bone marrow punch, and liver biopsy showed abundant acid-fast bacilli. Endoscopic findings on colonoscopy were multiple polypoid lesions approximately 5 mm in size in the transverse and sigmoid colon.

**Figure 1:**
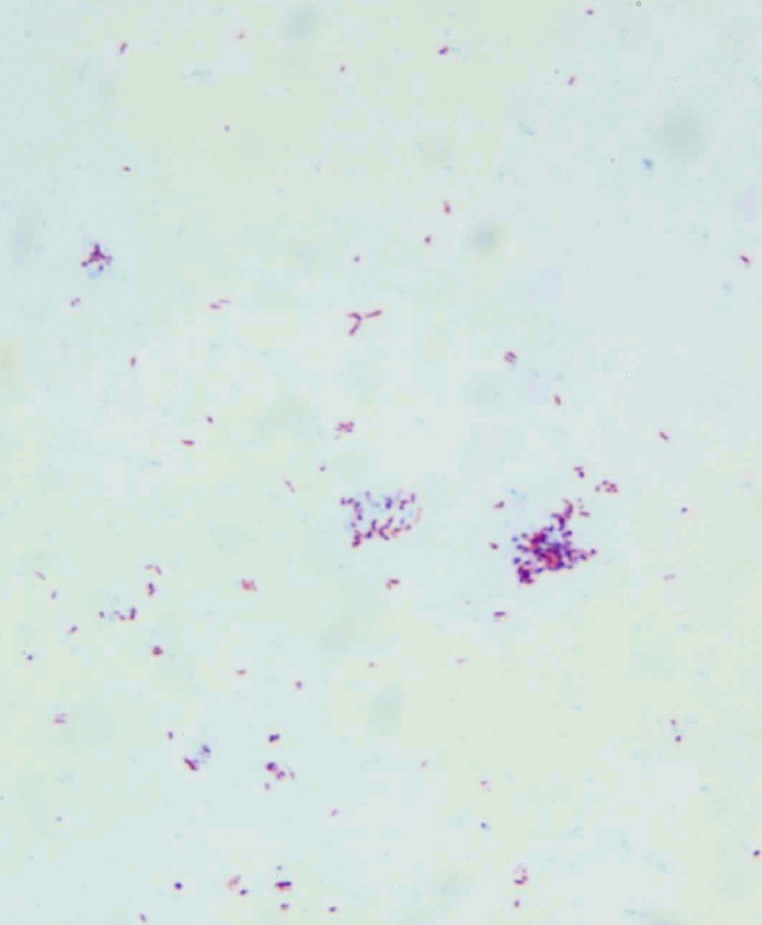
Ziehl-Neelsen–stained micrograph of *Mycobacterium avium* subsp. *paratuberculosis* colonies growing on mycobactin-supplemented Middlebrook agar.

Microbiologic analyses included culture for mycobacteria (liquid media: BACTEC 460TB or MGIT [Becton, Dickinson and Company, Cockeysville, MD] and solid media produced in-house, all media without supplementation of mycobactin) from at least 21 specimens (blood, urine, sputum, biopsy, feces) over a 3-year period. Of these, eight specimens (blood, feces, and biopsy) were positive for mycobacteria in liquid media after 6 to 16 weeks of incubation. Subcultures remained negative on Löwenstein-Jensen slants but after approximately 4 weeks became positive on mycobactin-supplemented Middlebrook slants with colorless dysgonic colonies. Microscopic examination of these colonies showed acid-fast bacilli (Figure 1).

**Figure 2:**
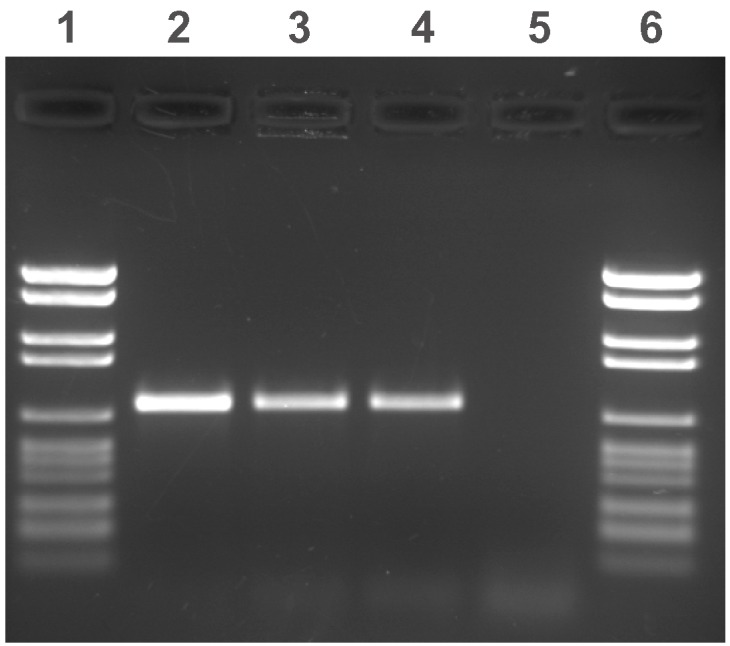
Agarose gel electrophoresis of amplified IS*900* fragments. Lanes 1 and 6: molecular weight marker (2176, 1766, 1230, 1033, 653, 517, 453, 394, 298, 234–220, 154 bp); lanes 2 and 3: two patient samples; lane 4: positive control (*Mycobacterium avium* subsp. *paratuberculosis* type strain); and lane 5: negative control *(Mycobacterium avium* strain).

For species identification, AccuProbe assays (Gen-Probe, San Diego, CA) for *M. avium* complex were performed on liquid media, all yielding strong positive results. However, repeated attempts to perform drug-susceptibility testing in the liquid BACTEC 460TB system were unsuccessful because of insufficient growth of the control. Since *M. avium* complex usually grows very well, the primary identification was questionable. Thus, polymerase chain reaction (PCR) for the amplification of a part of the mycobacterial gene coding for the ribosomal 16S RNA and additional sequencing was performed from two positive cultures (7). The resulting sequence was compared with those stored in the International Nucleotide Sequence Database (8), showing the signature sequence of *M. avium*/*M. paratuberculosis*, which is identical for both species and confirmed the AccuProbe results. For further differentiation between *M. avium* and MAP, PCR targeting the insertion sequence IS*900* (primer: IS*900*-1: 5´ TGTTCGGGGCCGTCGCTTAG; IS*900*-2: 5´-CGTTCCAGCGCCGAAAGTAT), which is present only in MAP strains (9), was done with the two most recent positive cultures. This assay showed clearly positive results from the two cultures tested and the MAP type strain, while the *M. avium* strains remained negative (Figure 2).

Because acid-fast bacilli were identified in biopsy specimens, treatment was started with ethambutol, ciprofloxacin, clarithromycin, and rifabutin. Initially, no clinical improvement was observed, and the patient’s weight loss and daily fever of 39C°–40°C continued. When ciprofloxacin was replaced with levofloxacin, progression of the infection appeared to stop. However, the patient died from cardiorespiratory failure.

## Conclusions

We describe the case of an HIV-infected patient who had a severe mycobacterial disorder thought to be caused by *M. avium* complex. Because growth was insufficient for susceptibility testing, the presence of MAP was assumed; however, the assumption was made after 2 years, because of difficulties in isolating MAP from human specimens (e.g., blood) in media not thought to enable its growth. Finally, the demonstration of the insertion sequence IS*900*, an assay not routinely performed in human diagnostic laboratories like ours, confirmed this hypothesis.

MAP isolated from human specimens has not yet been demonstrated by routine techniques. Several studies have reported the presence of MAP DNA in association with Crohn disease, although culture confirmation remains rare in these patients (5,6).

In the case we describe, mycobacterial growth could be detected in liquid media in 8 of 21 specimens, all confirmed as *M. avium* complex/*M. paratuberculosis*. However, because of the limited growth, we assume the presence of MAP even in those specimens not tested by IS*900* PCR. These results indicate that MAP can grow to a limited extent in routine liquid media without mycobactin supplementation, at least if present in high amounts in the specimen.

Susceptibility testing of the isolated strains could not be performed because of insufficient growth. Reports on susceptibility testing of MAP are rare, yet data obtained by a luciferase-based susceptibility assay (10) indicate susceptibility at least to clarithromycin and rifabutin, which were included in therapy. However, the patient’s response to treatment was not clearly positive and may have been hampered by his general poor health. This report suggests a pathogenic role of MAP for immunocompromised patients, raising the question of whether this strain so far has not been detected because of its limited growth, whether it has been misidentified as *M. avium,* or whether its occurrence in infections is low. However, herd prevalence of bovine paratuberculosis has been reported to range from 7% to 55% in Europe and to reach approximately 40% in stocks of >300 animals in the United States (4). Thus, consumption of inadequately pasteurized dairy products may be a possible risk for infection, especially for immunocompromised patients.
